# The Emergence of Tropical Medicine in France

**DOI:** 10.3201/eid2107.150521

**Published:** 2015-07

**Authors:** C.J. McKnight

**Affiliations:** Centers for Disease Control and Prevention, Atlanta, Georgia, USA

**Keywords:** tropical medicine, emergence of tropical medicine, disease theories, France, colonial medicine, naval medicine, provincial medical schools

The idea of naval medicine as a specific and discrete art is richly illustrated in The Emergence of Tropical Medicine in France, Michael Osborne’s historical account of French colonial medicine. For expanding European empires, the nineteenth century was a time when theories of tropical disease evolved as responses to distinct challenges on ships, in colonies, and in home ports. In France, a system of provincial medical schools was built by the navy in the port cities of Brest, Rochefort-sur-Mer, Toulon, and Bordeaux. Each faculty held to and taught their own system of medical knowledge retained within the regional boundaries. As described in this book, beliefs on causality and therapeutic options remained divided among the discrete spheres among institutions. The lack of accepted curricula seems a distant reminder of the many gains made before evidence-based medicine. To enrich the perspective, most of the book’s content is set in advance of the study of medical geography, which settled some longstanding misconceptions about ethnicity, location, and disease. Confusion reigned in colonial settings because of the similarity of causes implied during outbreaks of yellow fever, cholera, plague, typhus, and typhoid fever. 

This book does not address the scientific advancements on infectious etiologies; rather, it provides the context for French innovation within colonial functionaries, clashing ideologies, and commercial considerations. Medical training played a pivotal role in French colonial activity, as in Madagascar in 1895, when expeditionary forces were decimated by the thousands from malaria. While the prevailing belief was that tilled and swampy land caused the illness, that belief was overturned, by persons with medical training, in favor of insect bites. Success in Madagascar, as well as other overseas colonies, depended upon knowing disease cycles and managing interaction in the human populations. 

This book is a worthwhile investment for those interested in historical narratives on tropical medicine previously unavailable in the English language. Naval physicians like Charles-Adolphe Maher did remarkable studies while touring the tropics. In 1823, after having studied at Rochefort, he spent 2 years voyaging and encountered yellow fever outbreaks in Havana and Veracruz. Within the confines of his ship, Maher carefully compared the spectrum of symptoms. His conclusion on intermittent fever being a variety of malaria was far from correct, yet Mahler did initiate brave comparisons of therapies among patients, albeit with bloodletting and dietary privations. Maher’s lifelong findings on medical statistics, Statistique Medicale de Rochefort, first published in 1874 and recently reprinted, recount the lively experiences of Mahler and many other persons investigating *médicine exotique*. Osborne’s book provides key insight regarding influential persons who revolutionized notions of disease, recognizing their contributions as harbingers for the vast developments to follow in the twentieth century.

